# Synthesis and Quantitative Structure-Property Relationships of Side Chain-Modified Hyodeoxycholic Acid Derivatives

**DOI:** 10.3390/molecules180910497

**Published:** 2013-08-30

**Authors:** Paola Sabbatini, Paolo Filipponi, Roccaldo Sardella, Benedetto Natalini, Roberto Nuti, Antonio Macchiarulo, Roberto Pellicciari, Antimo Gioiello

**Affiliations:** 1 TES Pharma S.r.l., Via P. Togliatti, 20, Loc Taverne, 06073 Corciano, Italy; E-Mails: paola@chimfarm.unipg.it (P.S.); padulman@hotmail.com (R.N.); rpellicciari@tespharma.com (R.P.); 2 Dipartimento di Chimica e Tecnologia del Farmaco, Università degli Studi di Perugia, Via del Liceo, 1, 06123 Perugia, Italy; E-Mails: paolof@chimfarm.unipg.it (P.F.); roccaldo@chimfarm.unipg.it (R.S.); benedetto.natalini@unipg.it (B.N.); antonio@chimfarm.unipg.it (A.M.)

**Keywords:** bile acids, structure-property relationships, critical micellization concentration

## Abstract

Bile acids have emerged as versatile signalling compounds of a complex network of nuclear and membrane receptors regulating various endocrine and paracrine functions. The elucidation of the interconnection between the biological pathways under the bile acid control and manifestations of hepatic and metabolic diseases have extended the scope of this class of steroids for *in vivo* investigations. In this framework, the design and synthesis of novel biliary derivatives able to modulate a specific receptor requires a deep understanding of both structure-activity and structure-property relationships of bile acids. In this paper, we report the preparation and the critical micellization concentration evaluation of a series of hyodeoxycholic acid derivatives characterized by a diverse side chain length and by the presence of a methyl group at the alpha position with respect to the terminal carboxylic acid moiety. The data collected are instrumental to extend on a quantitative basis, the knowledge of the current structure-property relationships of bile acids and will be fruitful, in combination with models of receptor activity, to design and prioritize the synthesis of novel pharmacokinetically suitable ligands useful in the validation of bile acid-responsive receptors as therapeutic targets.

## 1. Introduction

Bile acids (BAs) and their derivatives are important endogenous steroids endowed with pleiotropic activities [1,2,3,4]. Beyond their primary digestive function in dietary lipid absorption, BAs are the crucial players of a complex receptor network regulating several patho-physiological states. Indeed, BAs were found to be ligands of nuclear hormone receptors, such as the Farnesoid X Receptor (FXR) [5,6,7], Vitamin D Receptor (VDR) [8], Pregnane X Receptor (PXR) [9], and of membrane receptors including the G-protein coupled receptor TGR5 [10]. Consistent with these findings, BAs are now considered versatile signalling compounds able to control their own biosynthesis, transport and metabolism through the gene expression modulation of several enzymes and proteins [1,2,3,4]. Moreover, recent reports have shown that BAs can influence cholesterol and lipid homeostasis, glucose levels, energy consumption and the immune response. As the consequence, BAs-mediated pathways have rapidly become attractive therapeutic targets for the management of hepatic and metabolic disorders [11,12,13].

In this scenario, the search for new and effective BA derivatives to be used for the clarification of the functional roles and therapeutic potential of BA receptors has represented an active field in medicinal and synthetic chemistry [14]. Indeed, naturally occurring BAs are endowed with low potency and selectivity, are systemically metabolized and, when they are administered in high concentrations, can be toxic. All these “non-receptor-based” properties make them unfit to be employed in *in vivo* investigations thus prompting the development of new suitable derivatives endowed with better drug-like profiles.

Two major patterns of modification were elaborated around the BA scaffold, namely, those affecting the B ring of the steroid nucleus and those relating to the side chain [15,16,17,18,19,20,21,22,23]. These studies have revealed that even minor modifications designed to obtain a desired biological activity, can also significantly affect the physico-chemical properties, metabolic behaviour, distribution within different districts and tissues, and, most importantly, the cytotoxicity profile of the new analogues with respect to the parent natural BA [24,25,26]. In particular, the presence and/or different orientations of the hydroxyl groups, modifications of the steroidal nucleus and side chain, induce different conformational arrangements of the BA that can strongly influence the physico-chemical characteristics directly linked to detergency.

Detergency is one the most important properties of BAs, being essential for the absorption and digestion of lipid-soluble nutrients, by mediating their solubilisation into micelles, as well as being the potential cause of BA cytotoxicity. Detergency is strictly related to the hydrophobic/hydrophilic balance (HHB) of BAs and can be evaluated by the determination of critical micellization concentration (CMC), which is considered a key parameter that needs to be addressed in the preliminary phase of compound characterization. Molecules with high CMC values are poor detergents and therefore poorly toxic to biological membranes.

**Figure 1:**
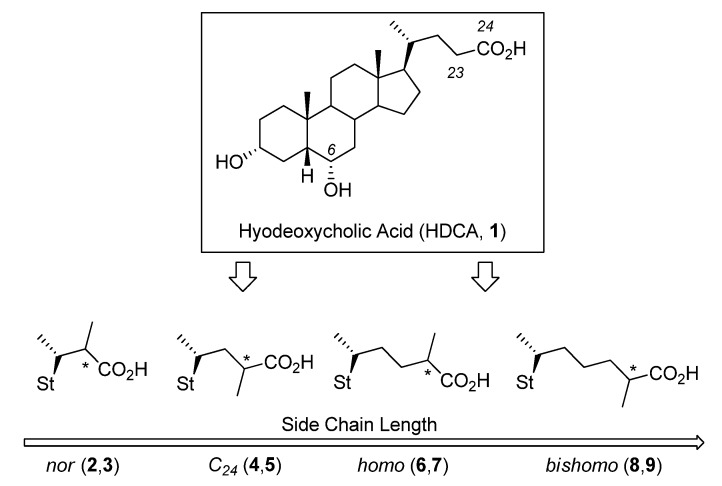
Synthesis of HDCA Derivatives **2**–**9**.

Based on these considerations and following our interest in BA research [15,16,18,19,20,21,22], in this paper, we investigate the effect of BA side chain modifications with respect to their capacity to generate micelles. In particular, we report the synthesis, CMC evaluation and structure-property considerations of a series of hyodeoxycholic acid (HDCA) derivatives characterized by a different side chain length and by the presence of a methyl group at the alpha position of the carboxylic acid tail (Figure 1). HDCA (**1**) was selected as the model compound for this study because of its peculiar physico-chemical and metabolic profile. HDCA (**1**) is a secondary BA formed by bacterial C-7 alpha dehydroxylation of hyocholic acid and ω-muricholic acid (pig, rat) or by C-6 hydroxylation of lithocholic acid (human). HDCA (**1**) is endowed with a safer detergency (lower CMC) than chenodeoxycholic acid (CDCA) and cholic acid (CA) (Table 1), and a unique metabolism among common natural BAs in undergoing C-6 glucuronidation in man, but not in experimental animal models. Moreover, the great synthetic versatility of the compound documented by the numerous works where it was employed as a precursor of bioactive steroids, makes HDCA (**1**) a valuable alternative to CDCA and CA (Table 1) for the development of pharmacokinetically suitable BA-based chemical tools for exploring the biological relevance and therapeutic application of BA-mediated receptors.

**Table 1:** CMC values of the investigated HDCA derivative sodium salts with respect to CDCA and CA.

Entry	Compound	Estimated CMC (mM)	Determined CMC (mM)	CHI
1	CDCA	8.0	7.0	0.87
2	CA	12.0	13.0	0.83
3	HDCA (**1**)	15.0	15.0	0.81
4	**3**	24.2	24.0	0.76
5	**4**	18.1	17.6	0.79
6	**5**	10.2	-	0.85
7	**6**	8.7	7.8	0.86
8	**7**	7.5	-	0.88
9	**8**	6.8	-	0.89
10	**9**	6.3	6.4	0.90

## 2. Results and Discussion

### 2.1. Synthesis

**Scheme 1:**
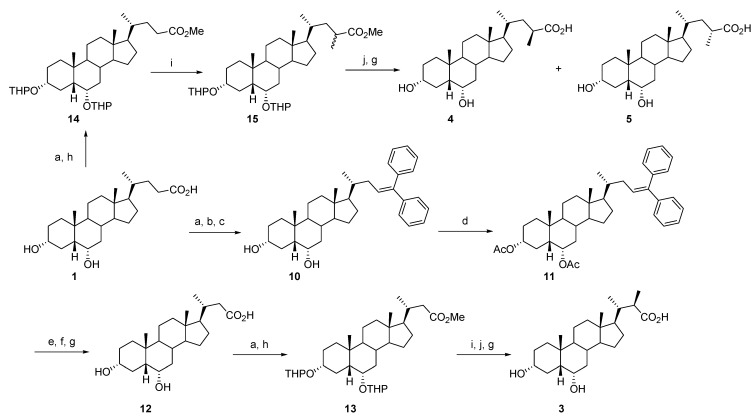
Synthesis of HDCA-modified derivatives **2**, **4**–**5**.

**Scheme 2:**
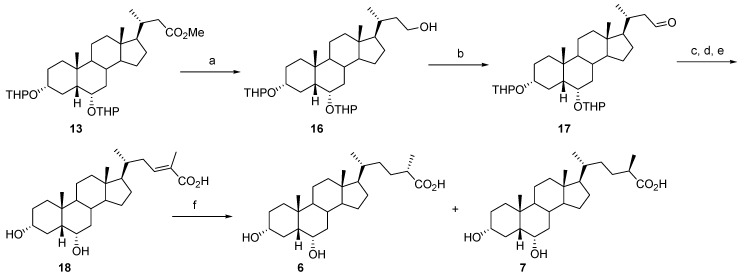
Synthesis of C_24_-methyl HDCA derivatives **6**–**7**.

Compounds **8** and **9** were prepared as previously reported [27]. All the other derivatives were synthesized as illustrated in Scheme 1 and Scheme 2.

*Reagents and conditions*: (**a**) MeOH, *p-*TSA, ultrasound; (**b**) PhMgBr, THF, reflux; (**c**) HCl, EtOH, 60 °C;(**d**) Ac_2_O, Et_3_N, CH_2_Cl_2_, r.t.; (**e**) O_3_, CH_2_Cl_2_, then NaBH_4_, MeOH, −78 °C; (**f**) Jones reagent, acetone, 0 °C; (**g**) NaOH, MeOH, r.t.; (**h**) *p-*TSA, DHP, dioxane, r.t.; (**i**) LDA, MeI, THF, −78 °C; (**j**) MeOH/HCl 37%, r.t.

*Reagents and conditions*: (**a**) LiAlH_4_, THF, r.t.; (**b**) Dess Martin periodinane, CH_2_Cl_2_, r.t.; (**c**) (1-(ethoxycarbonyl)ethyl)triphenylphosphonium bromide, KO*t-*Bu, THF, r.t then reflux; (**d**) MeOH/HCl 37%, r.t.; (**e**) NaOH, MeOH, r.t.; (**f**) PtO_2_, H_2_,MeOH.

Thus, HDCA (**1**) was transformed into the corresponding ester by treatment with catalytic amount of *p*-toluenesulfonic acid (*p-*TSA) in MeOH under ultrasound heating conditions. The ester intermediate was reacted with an excess of PhMgBr in refluxing THF to give the Grignard adduct, which was readily converted into the olefin **10** by acidic dehydration (HCl/EtOH, 98% yield). The protection of the hydroxy groups was performed with Ac_2_O and Et_3_N in CH_2_Cl_2_ at room temperature to furnish the 3α,6α-diacetylated derivative **11** in quantitative yield. Ozonolysis, reduction of the aldehyde thus formed with NaBH_4_ in MeOH, followed by Jones oxidation and basic hydrolysis (NaOH/MeOH) gave the desired 3α,6α-dihydroxy-24-nor-5β-cholan-23-oic acid (**12**), in 69% yield. Esterification and protection of the hydroxy groups at the C-3 and C-6 position with 3,4-dihydro-2*H*-pyran in the presence of catalytic *p-*TSA in dioxane at room temperature, afforded methyl 3α, 6α-tetrahydropyranyloxy-24-nor-5β-cholan-23-oate (**13**), in 70% yield after chromatographic purification (Scheme 1). Compound **13** was then methylated at the C-22 position by reaction with LDA and methyl iodide at −78 °C in freshly distilled THF. Finally, acidic (HCl/MeOH) and basic (NaOH/MeOH) hydrolysis afforded 22(*R*)-methyl-3α,6α-dihydroxy-23-nor-5β-cholan-23-oic acid (**3**) as a single isomer, in 86% yield. The stereochemical outcome of the alkylation reaction can be explained considering the specific spatial disposition of the enolate ester in the preferred ‘H-eclipsed’ conformation, that forces the methyl iodide to approach from the less hindered *R*-prochiral face of the lithium enolate [28]. Notably, when the same reaction was conducted on the C_24_ analog **14**, the corresponding C_23_ epimers **4** and **5** were isolated in nearly 1:1 ratio, after alkaline/acidic hydrolysis and purification by medium pressure liquid chromatography (**4**: 37%, **5**: 33%; Scheme 1). In this case, it can be speculated that the presence of an additional carbon atom increases the degree of conformational freedom of the side chain, making the diastereotopic faces of the enolate similarly reactive.

Compounds **6** and **7** were synthesized as depicted in Scheme 2. The reduction of the intermediate **13** with LiAlH_4_ in THF at room temperature followed by Dess Martin periodinane oxidation gave 3α,6α-bis(tetrahydropyranyloxy)-24-nor-5β-cholan-23-al (**17**), in quantitative yield. The reaction of **17** with (1-(ethoxycarbonyl)ethyl)triphenylphosphonium bromide, with KO*t-*Bu used as the base, in refluxing THF furnished the desired 3α,6α-dihydroxy-24-methyl-5β-homochol-23-en-25-oic acid (**18**, 70%) after acid/base-induced hydrolysis. Hydrogenation of the C-23/C-24 double bond was performed using PtO_2_ as catalyst in MeOH at room temperature. The resulting C_24_(*S*)- and C_24_(*R*)-derivatives **6** and **7** were obtained in 94% yield and a 6:4 ratio, respectively. The absolute configuration assignment to epimers was based upon the previously reported single-crystal X-ray structure, ^13^C-NMR comparison and HPLC retention considerations [20].

### 2.2. Chromatographic CMC Estimation

Unlike conventional ionic surface active agents, BA aggregation materializes over a broad concentration range, through a “stepwise aggregation process” [29,30]. This feature well justifies the more realistic concept of a “noncritical multimer concentration” [31,32], which encodes for the non-cooperative and continuous association by the BS monomers. Owing to the polydispersity of the BA aggregates [33,34,35,36], many of the methods proposed for determining the start of the aggregation event (that is the CMC) do not allow one to sensitively detect subtle variations of the physico-chemical state of the system. Therefore, the CMC values of these steroidal compounds are markedly related to the method selected for determining them [31,32].

Spectrophotometry in the absence of exogenous probe molecules has proved to be effective in revealing the formation of low aggregation number BA assemblies [37,38]. The CMC can be identified by monitoring the profile of variation of some intrinsic property of the system (such as *Abs*_max_ around 195–200 nm of the main characteristic UV band) with the surfactant concentration [37].

The HHB of common BAs is linearly related to their aqueous CMC [39] and is importantly affected even by minor modifications in their molecular structure. Such slight differences are advantageously emphasized in a reversed phase (RP) setting, which makes the chromatographic approach the method of choice for HHB evaluation.

In a recent work [38], we produced evidence of a strict correlation between spectrophotometric CMC and chromatographic hydrophobicity index (CHI) values, obtained by running gradient elution RP-HPLC analyses. In the work, 14 unconjugated C_24_ BAs featuring a different number, position and orientation of hydroxy groups, as well as other substituents on the steroidal backbone and side chain, were selected to build up a statistically relevant pCMC *vs*. CHI calibration curve [38]. For a given compound, the CHI value can be thus used as a measure of its peculiar HHB. Moreover, to rely upon CHI values excludes a number of problems which are typical of monocratic methods. In particular, with suitable gradient elution profiles, retention data from compounds having markedly different hydrophobicities can be directly compared without the necessity to run analyses under different conditions (different column lengths and/or eluent compositions), which could ultimately result in difficult cross-correlation procedures.

In the present work, the same mathematical model has been used to estimate the CMC of the investigated HDCA derivative sodium salts (Table 1). The suitability of the previously established correlation model, even for BSs endowed with different side chain lengths, has been confirmed by carrying out spectrophotometric CMC determinations on a selected sub-set of compounds (**3**, **4**, **6** and **9**, Table 1). Notably, this chromatographic approach to CMC evaluation has a number of distinctive advantages over all the other methods: it can be automated, only a small amount of sample is required, and impurities do not disturb the measurement results.

### 2.3. Discussion

The complex nature of BAs requires that their receptor potency and selectivity, pharmacological actions as well as their physico-chemical properties be well-defined. In this regard, the understanding of the relationships between their structure and properties, and, in particular, the evaluation of their detergency and attitude to generate micelles in bile, are the crucial factors which may facilitate, or limit, their advancement in preclinical and clinical settings. These peculiar features of BAs can be estimated by determining the CMC, a relevant parameter due to its physio-pathological implications. 

It is well known that the CMC value of BAs is strongly influenced by the number, position and stereochemistry of polar and apolar groups present on the steroidal framework [31,40]. In general, it can be assumed that for the nucleus the key factor influencing the BA self-association to form micelles, is essentially the contiguity of the hydrophobic β-surface: any decrease in this area raises the CMC. Indeed, a BA characterized by hydrophilic groups at both sides of the steroid has a lower propensity to form micelles even at high monomeric concentrations. Although less investigated, there is evidence that modifications of the BA side chain can also influence the extent of this phenomenon [31]. In particular, it was reported that the CMC increases with the shortening of the side chain [41], while the substitution at the alpha position to the carboxylic acid tail may cause a strong variation of the BA micellar attitude. This notion was confirmed by the determination of the CMC of all four stereoisomers of 3α,7β-dihydroxy-22,23-methylene-5β-cholan-24-oic acid showing different values accordingly to the configuration of the C-22 and C-23 stereocenters [42].

**Figure 2:**
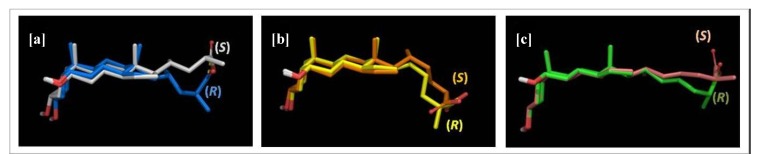
Overlapping of the calculated global minimum conformations of the diverse couple of epimers. (**a**) C_24_(*S*) and C_24_(*R*) epimers **4** and **5**. (**b**) C_25_(*S*) and C_25_(*R*) epimers **6** and **7**. (**c**) C_26_(*S*) and C_26_(*R*) epimers **8** and **9**.

In this work, we investigated the effect on the micellization behaviour of HDCA derivatives **3**–**9** prepared by side chain shortening and elongation, combined with the insertion of a methyl group in the alpha position to the carboxylic acid moiety in both (*R*)- and (*S*)-configurations (Table 1). Thus, it was found that the CMC value progressively decreases with the elongation of the side chain, in line with previous observations [31]. Interestingly, a strong influence on the aggregation propensity was found to be exerted by the presence and optical configuration of the methyl group (Table 1, entries 3, 6–7): a different conformational asset can be indeed envisaged for each couple of epimers causing a distinct facial aggregation behaviour. In Figure 2, the superimposition of the obtained global minimum conformations for each couple of epimers clearly shows the different side chain disposition of the epimers. It is worth noting that the different ability to form micelles between the parent (*R*)- and (*S*)*-*epimers decreases with the elongation of the side chain. This effect seems to be more evident for the C_24_ derivatives **4** and **5** that are characterized by a greater difference in the relative CMC values (Table 1, entries 6, 7), while it is reduced for the longer side chain analogs **6**–**9** (Table 1, entries 7–10). This tendency was quantitatively estimated by calculating the root-mean-square deviation (RMSD) value, which measure the average distance between the heavy atoms of the epimer side chain. The higher is the RMSD value, the bigger is the difference between the conformational aspect of the molecule, and, in particular, of the side chain. Thus, the RMSD value is 2.23 Å for compounds **4** and **5** (C_24_ epimers), 1.92 Å for compounds **6** and **7** (C_25_ epimers) and 1.65 Å for compounds **8** and **9** (C_26_ epimers).

**Figure 3:**
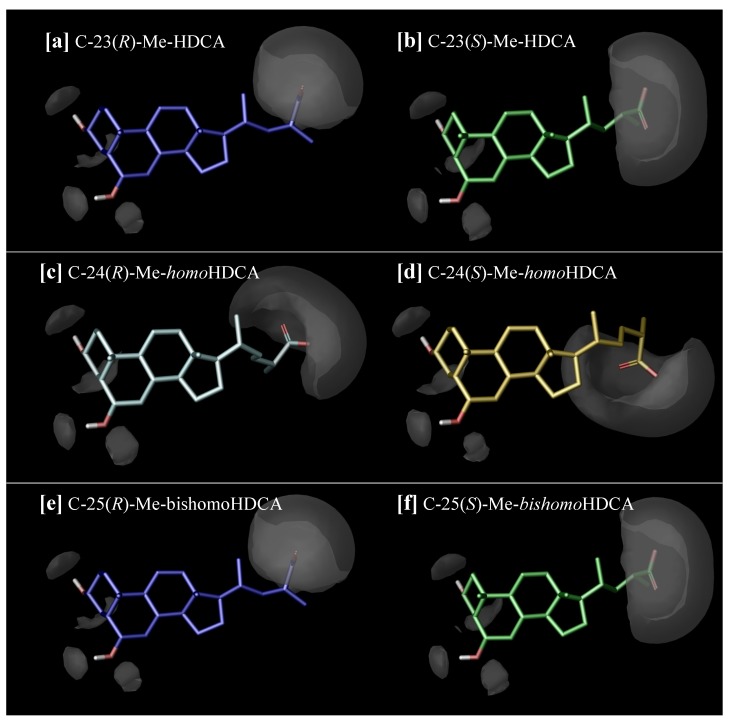
Disposition of the favourable hydrophilic volumes in (**a**) C-23(*R*)-methyl HDCA (**5**); (**b**) C-23(*S*)-methyl HDCA (**4**); (**c**) C-24(*R*)-methyl homoHDCA (**7**); (**d**) C-24(*S*)-methyl homoHDCA (**6**);(**e**) C-25(*R*)-methyl bishomoHDCA (**9**); (**f**) C-25(*S*)-methyl bishomoHDCA (**8**).

Moreover, as a general trend, the (*R*)-epimers **5**, **7**, **9** tend to better form micelles with respect to the parent (*S*)-analogs **4**, **6**, **8**. As illustrated in Figure 3, when the methyl is in the (*S*)-configuration, the carboxy group points toward the beta face of the molecule, resulting in a reduction of the hydrophobic area and the consequent increase of the CMC value. On the other hand, (*R*)-methyl group forces the carboxylate toward the hydrophilic alpha face, thus apparently not affecting the extension of the hydrophobic area. This phenomenon is strongly evident in the C_24_ epimers **4** and **5** (Figure 2[a,b]) and, although to a less extent, can be observed for both the *homo* and *bishomo* epimers **6**–**9** (Figure 2[c–f]).

## 3. Experimental

### 3.1. General Methods

All reagents were commercially available unless otherwise noted. All reactions were carried out in dried glassware under a dry nitrogen atmosphere. The final products were purified by chromatography on silica gel (70–230 mesh). TLC was performed on aluminium backed silica plates (silica gel 60 F254). Spots on TLC were visualized by using UV and by staining and warming with phosphomolybdate reagent (5% solution in EtOH). All the reactions were performed using distilled solvent. ^1^H-NMR spectra were recorded at 200 and 400 MHz, ^13^C-NMR spectra were recorded at 50.3 and 100.6 MHz, respectively, using the solvents indicated below. Chemical shifts are reported in parts per million. The abbreviations used are as follows: s, singlet; d, doublet; t, triplet; q, quartet; br s, broad singlet. Melting points were determined with an electrothermal apparatus and are uncorrected. Compounds **8** and **9** were prepared as previously reported [27].

### 3.2. Synthesis of Compounds ***3**–**7***

*24,24-Diphenyl 3α,6α-dihydroxy-5β-cholan-23-ene* (**10**). To a solution of HDCA (**1**, 13.65 g, 34.82 mmol) in MeOH (200 mL), *p-*TSA (0.67 g, 3.48 mmol) was added and the resulting mixture was sonicated at 25 °C for 3 h. The solvent was then evaporated *in vacuo*, the crude was dissolved in CHCl_3_ (200 mL), washed with NaHCO_3_ss (150 mL), brine (150 mL), dried over Na_2_SO_4_ and evaporated under reduced pressure. The ensuing methyl ester was dissolved in dry THF (130 mL) and refluxed. To this boiling solution PhMgBr (208 mL, 1 M in THF) was quickly added dropwise. The gelatinous mixture obtained was refluxed for 16h, then cooled and treated with cyclohexane (300 mL) for 2 h. The solid thus obtained was filtered off, washing the filter with additional 200 mL of cyclohexane. The solid was then dissolved in HCl 1 N (300 mL) and CH_2_Cl_2_ (300 mL), the phases were separated, the organic layer was dried over Na_2_SO_4_ and evaporated under reduced pressure. The oily crude was dissolved in EtOH (200 mL) and HCl 37% (10 mL) and warmed to 60 °C for 2 h. The solvent was evaporated and the residue was dissolved in H_2_O (200 mL) and extracted with CH_2_Cl_2_ (3 × 100 mL). The combined organic layers were washed with brine (200 mL), dried over Na_2_SO_4_ and evaporated under reduced pressure. The solid thus obtained was treated with 10% NaOH in MeOH (100 mL) at room temperature for 14 h, in order to hydrolyze the unreacted ester. The solvent was evaporated, dissolved in H_2_O (200 mL) and extracted with EtOAc (3 × 100 mL). The water phase was acidified to pH = 3 and extracted with EtOAc (2 × 100 mL), to get, after evaporation of the solvent, 4.15 g (10.50 mmol) of unreacted HDCA (**1**). The combined organic layers were washed with brine (200 mL), dried over Na_2_SO_4_ and evaporated to dryness, to obtain the desired olefin **10** as white solid (12.14 g, 23.71 mmol, conversion yield: 98%). ^1^H-NMR (CDCl_3_, 400 MHz) δ: 0.65 (3H, s, 18-C*H_3_*), 0.90 (3H, s, 19-C*H_3_*), 0.96 (3H, d, *J =* 6 Hz, 21-C*H_3_*), 3.7–3.61 (1H, m, 3-C*H*), 4.02–4.05 (1H, m, 6-C*H*), 6.11 (1H, t, *J =* 7 Hz, 23-C*H*), 7.14–7.39 (10H, m, ArH). ^13^C-NMR (CDCl_3_, 100.6 MHz) δ: 11.97, 19.01, 20.68, 23.45, 24.18, 28.10, 29.18, 30.09, 34.77, 35.53, 35.89 (2x), 35.93, 36.90, 39.80, 39.83, 42.80, 48.38, 56.05, 56.10, 68.00, 71.50, 126.22, 127.05 (2×), 127.99 (4×), 128.23, 129.38, 129.95 (2×), 140.34, 142.17, 143.82.

*24,24-Diphenyl 3α,6α-diacetoxy-5β-cholan-23-ene* (**11**). To a solution of olefin **10** (12.0 g, 23.43 mmol) in dry CH_2_Cl_2_ (100 mL) Et_3_N (19.6 mL, 140.62 mmol) and Ac_2_O (11.06 mL, 117.18 mmol) were added and the resulting mixture was refluxed for 2 h. The mixture was allowed to cool to room temperature then was poured into aqueous HCl 1 N (150 mL) and extracted with CH_2_Cl_2_ (3 × 80 mL). The combined organic layers were washed with NaHCO_3_ss (150 mL), H_2_O (150 mL), brine (150 mL), dried over Na_2_SO_4_ and evaporated under reduced pressure, obtaining the desired acetylated compound **11** as whitish solid (13.83 g, 23.19 mmol, 99%). ^1^H-NMR (CDCl_3_, 400 MHz) δ: 0.64 (3H, s, 18-C*H_3_*), 0.94-0.97 (6H, m, 19-C*H_3_* + 21-C*H_3_*), 2.00 (3H, s, OAc), 2.03 (3H, s, OAc), 4.68–4.72 (1H, m, 3-C*H*), 5.11–5.18 (1H, m, 6-C*H*), 6.11 (1H, t, *J =* 6.3 Hz, 23-C*H*)), 7.13–7.36 (10H, m, ArH). ^13^C-NMR (CDCl_3_, 50.3 MHz) δ: 11.84, 18.89, 20.50, 21.24 (2×), 23.11, 23.93, 26.06, 26.26, 27.95, 31.11, 34.43, 34.86, 35.85 (2×), 36.69, 39.66 (2×), 42.70, 45.17, 55.92 (2×), 70.78, 73.50, 126.58, 126.93 (2×), 127.89 (4×), 128.59, 129.25, 129.83 (2×), 140.21, 142.13, 142.78, 170.18 (2×).

*3α,6α-Dihydroxy-24-nor-5β-cholan-24-oic acid* (**12**). A solution of **11** (13.70 g, 22.98 mmol) in CH_2_Cl_2_ (200 mL) kept at −78 °C was ozonized until the deep blue colour was persistent for 30 min. At this point the ozonization was interrupted and the blue deep solution was swept with nitrogen to purge the dissolved ozone. The solution was then warmed at room temperature, evaporated to dryness, dissolved in distilled MeOH (100 mL) and treated with NaBH_4_ (3.49 g, 91.92 mmol) at room temperature overnight. The reaction was then quenched with H_2_O (100 mL) and volatiles were removed under reduced pressure. The crude was dissolved in CH_2_Cl_2_ (100 mL), washed with HCl 3 N (100 mL), H_2_O (100 mL), brine (100 mL), dried over Na_2_SO_4_ and evaporated under reduced pressure. A fresh made Jones reagent (10.0 mL) was added dropwise to a stirred solution of the alcohol thus obtained in acetone (100 mL) at 0 °C and the mixture was stirred at room temperature for 2 h. Methanol (40 mL) was then added, the solvent was evaporated under reduced pressure, dissolved in H_2_O (100 mL) and extracted with EtOAc (2 × 60 mL). The combined organic layers were dried over Na_2_SO_4_ and evaporated under reduced pressure The oily residue was treated with NaOH 10% in MeOH (138 mL) at room temperature for 16 h, then the solvent was removed *in vacuo*. The crude was dissolved in H_2_O (500 mL) and extracted with a mixture of Et_2_O/EtOAc (9:1, 2 × 200 mL). The water phase was then treated with HCl 37% until pH= 2, then extracted with CH_2_Cl_2_ (2 × 200 mL). The combined organic phases were washed with brine (300 mL), dried over Na_2_SO_4_ and evaporated to dryness to obtain the desired nor-HDCA **12** as white solid (6.10 g, 15.87 mmol, 69%). ^1^H-NMR (CDCl_3_ + MeOD, 400 MHz) δ: 0.58 (3H, s, 18-C*H_3_*), 0.80 (3H, s, 19-C*H_3_*), 0.90 (3H, d, 21-C*H_3_*), 3.46–3.48 (1H, m, 3-C*H*), 3.89–3.93 (1H, m, 6-C*H*). ^13^C-NMR (CDCl_3_ + MeOD, 100.6 MHz) δ: 11.83, 19.22, 20.55, 23.33, 23.99, 28.08, 28.59, 29.56, 33.54, 34.23, 34.66, 35.37, 35.73, 39.63 (2x), 41.31, 42.73, 48.16, 55.87, 56.01, 67.62, 71.05, 176.42.

*Methyl 3α,6α-bis(tetrahydropyranyloxy)-24-nor-5β-cholan-23-oate* (**13**) To a solution of norHDCA **12** (5.8 g, 15.34 mmol) in MeOH (200 mL), *p-*TSA (0.29 g, 1.53 mmol) was added and the resulting mixture was sonicated at 25 °C for 5 h. The solvent was then evaporated *in vacuo*, the crude was dissolved in CHCl_3_ (100 mL), washed with NaHCO_3_ss (100 mL), brine (100 mL), dried over Na_2_SO_4_ and evaporated under reduced pressure. The ensuing methyl ester was dissolved in dioxane (40 mL) and *p*-TSA (0.26 g, 1.40 mmol) and 3,4-DHP (19.2 mL, 210.46 mmol) was added dropwise in 2 h. At the end of the addition the mixture was poured into H_2_O (120 mL) and extracted with EtOAc (3 × 40 mL). The combined organic layers were washed with H_2_O (100 mL), brine (100 mL), dried over Na_2_SO_4_ and evaporated under reduced pressure. The yellow oily residue was purified by flash chromatography eluting with petroleum ether/EtOAC from 0 to 20%. The desired compound **13** was obtained as colourless oil as pure mixture of four diastereoisomers (4.86 g, 9.81 mmol, 70%). ^1^H-NMR (CDCl_3_, 400 MHz) δ: 0.62 (3H, s, 18-C*H_3_*), 0.86 (3H, s, 19-C*H_3_*), 0.92 (3H, d, *J =* 6.1 Hz, 21-C*H_3_*), 3.42–3.45 (2H, m, C*H_2_*-THP), 3.55–3.58 (1H, m, 3-C*H*), 3.61(3H, s, COO*CH_3_*), 3.83–3.88 (2H, m, C*H_2_*-THP), 3.95–3.98 (1H, m, 6-C*H*), 4.57–4.70 (2H, m, 2 × C*H*-THP).

*3α,6α-Dihydroxy-22(R)-methyl-24-nor-5β-cholan-23-oic acid* (**3**) To a solution of diisopropylamine (2.95 mL, 21.15 mmol) in THF (48 mL) at −78 °C *n*BuLi (2M in hexane, 10.1 mL) was added dropwise in 20 min. After 30 min a solution of **13** (1.5 g, 3.15 mmol) in THF (32 mL) was added in 45 min. After 1 h at −78 °C MeI (2.92 mL, 47.36 mmol) was added in 15 min. The reaction was slowly allowed to warm to room temperature. The reaction was then poured in H_2_O (100 mL) and extracted with EtOAc (3 × 60 mL). The combined organic layers were washed with brine (100 mL), dried over Na_2_SO_4_ and evaporated under reduced pressure. The brown oily residue was dissolved in MeOH (95 mL) and treated with HCl 3 N (5 mL) at room temperature overnight. The solvent was then removed *in vacuo*, the crude was added with H_2_O (80 mL) and extracted with CH_2_Cl_2_ (3 × 60 mL). The combined organic layers were washed with brine (100 mL), dried over Na_2_SO_4_ and evaporated under reduced pressure. The deprotected methyl ester thus obtained was dissolved in NaOH 10% in MeOH (5.75 mL) and refluxed overnight. The mixture was then allowed to cool to room temperature, dissolved in H_2_O (100 mL) and treated with HCl 3 N until pH = 2. The mixture was then extracted with EtOAc (3 × 70 mL), washed with H_2_O (100 mL), brine (100 mL), dried over Na_2_SO_4_ and evaporated to dryness. The crude was then purified by flash chromatography with CH_2_Cl_2_/acetone from 0 to 50%, obtaining pure **3** as a single isomer (1.06 g, 2.71 mmol, 86%). ^1^H-NMR (CDCl_3_ + MeOD, 400 MHz) δ: 0.68 (3H, s, 18-C*H_3_*), 0.84 (3H, d, *J =* 6.7 Hz, 24-C*H_3_*), 0.89 (3H, s, 19-C*H_3_*), 0.96 (3H, d, *J =* 7.0 Hz, 21-C*H_3_*), 3.54–3.59 (1H, m, 3-C*H*), 3.98–4.04 (1H, m, 6-C*H*). ^13^C-NMR (CDCl_3_ + MeOD, 100.6 MHz) δ: 8.26, 11.89, 14.15, 20.64, 23.36, 23.94, 27.66, 68.72, 29.70, 34.38, 34.77, 35.43, 35.79, 37.06, 39.72, 39.84, 41.67, 42.64, 48.24, 53.24, 56.15, 67.72, 71.15, 179.30.

*Methyl 3α,6α-bis(tetrahydropyranyloxy)-5β-cholan-24-oate* (**14**). To a solution of HDCA (**1**, 2.5 g, 6.4 mmol) in MeOH (100 mL), *p*TSA (0.121 g, 0.64 mmol) was added, and the mixture was sonicated for 90 min. The mixture was then concentrated under reduced pressure, and the resulting residue was diluted with CHCl_3_ (100 mL), washed with NaHCO_3_ss (3 × 80 mL), brine (50 mL), dried over Na_2_SO_4_, and concentrated under reduced pressure. The residue was then dissolved in dioxane (50 mL), and treated with *p-*TSA (0.121 mg, 0.64 mmol). 3,4-DHP (10.71 g, 127.4 mmol) was then added dropwise in 3 h and the resulting mixture was stirred for further 2 h at room temperature monitoring the reaction by TLC (petroleum ether/EtOAc 9:1). The mixture was quenched with H_2_O (50 mL) and extracted with EtOAc (3 × 40 mL). The combined organic layers were washed with brine (50 mL), dried over Na_2_SO_4_, and concentrated under reduced pressure. The resulting residue was purified by flash chromatography using petroleum ether/EtOAc to obtain **14** (2.6 g, 70%) as colourless oil. ^1^H-NMR (acetone-d_6_, 400 MHz) δ: 0.69 (3H, m, 18-C*H*_3_), 0.93–0.96 (6H, m, 19-C*H*_3_ + 21-C*H*_3_), 3.31–3.45 (3H, m, 3-C*H*, OC*H*_2_), 3.60 (3H, s, COOC*H_3_*), 3.68–4.06 (3H, m, 6-C*H*, OC*H*_2_), 4.68–4.76 (2H, m, 2 × OC*H*(CH_2_)O).

*3α,6α-Dihydroxy-23(S)- and 23(R)-methyl-5β-cholan-24-oic acid* (**4** and **5**). To a solution of diisopropylamine (1.23 mL, 8.7 mmol) in freshly distilled THF (35 mL) cooled at −78 °C and under N_2_ atmosphere, *n*BuLi (2.5 M in hexane, 3.2 mL, 8.0 mmol) was added dropwise. The reaction was stirred at −78 °C for 30 min and then a solution of **14** (2 g, 3.48 mmol) dissolved in freshly distilled THF (15 mL) was added dropwise, and the reaction was stirred at −78 °C for 90 min. Methyl iodide (2.25 mL, 34.8 mmol) was then added dropwise, and the solution was stirred at −78 °C for 60 min and slowly warmed to room temperature overnight. The mixture was then concentrated under reduced pressure, and the resulting residue was diluted with H_2_O (50 mL) and extracted with EtOAc (3 × 40 mL). The combined organic layers were then washed with brine (50 mL), dried over Na_2_SO_4_, and concentrated under reduced pressure. The residue was then treated with a solution of MeOH/HCl 37% (80 mL, 20:1 vol/vol) at 45 °C for 12 h. The mixture was concentrated under reduced pressure, and the resulting residue was diluted with H_2_O (50 mL) and extracted with EtOAc (3 × 40 mL). The combined organic layers were washed with brine (50 mL), dried over Na_2_SO_4_, and concentrate under reduced pressure. The resulting residue was refluxed with a solution of NaOH 2 M in MeOH (40 mL) at 45 °C over night. The mixture was then concentrated under reduced pressure, and the resulting residue was dissolved in H_2_O (50 mL), washed with *i*Pr_2_O (2 × 20 mL), acidified with HCl 3 N, and extracted with CHCl_3_ (3 × 40 mL). The combined organic layers were washed with brine (100 mL), dried over Na_2_SO_4_, and concentrate under reduced pressure. The resulting residue was purified by medium pressure chromatography (column: “RP-18 Lobar B”, MeOH/H_2_O) to give **4** (520 mg, 37%) and **5** (470 mg, 33%) as pure white solids. *3α,6α-Dihydroxy-23(S)-methyl-5β-cholan-24-oic acid* (**4**): mp: 255 °C. ^1^H-NMR (CD_3_OD, 400 MHz) δ: 0.67 (3H, s, 18-C*H*_3_), 0.90–0.99 (6H, m, 19-C*H*_3_, 21-C*H*_3_), 1.13 (3H, d, *J* = 6.9 Hz, 23-C*H*_3_), 2.55 (1H, m, 23-C*H*), 3.51 (1H, m, 3-C*H*), 4.01 (1H, m, 7-C*H*). ^13^C-NMR (CD_3_OD, 100.6 MHz) δ: 12.59, 19.07, 19.39, 21.90, 24.08, 25.26, 29.20, 29.99, 31.13, 35.56, 35.88, 36.17, 36.79, 36.92, 38.50, 41.30, 41.40, 42.18, 44.05, 49.05, 57.66, 58.19, 68.65, 72.38, 180.93. *3α,6α-Dihydroxy-23(R)-methyl-5β-cholan-24-oic acid* (**5**): mp: 199 °C. ^1^H-NMR (CD_3_OD, 400 MHz) δ: 0.70 (3H, s, 18-C*H*_3_), 0.92 (3H, s, 19-C*H*_3_), 0.94 (3H, d, *J* = 6.4 Hz, 21-C*H*_3_), 1.08 (3H, d, *J* = 6.9 Hz, 23-C*H*_3_), 2.44 (1H, m, 23-C*H*), 3.50 (1H, m, 3-C*H*), 4.00 (1H, m, 7-C*H*). ^13^C-NMR (CD_3_OD, 100.6 MHz) δ: 12.45, 16.89, 18.75, 21.91, 24.07, 25.27, 29.54, 30.00, 31.14, 35.19, 35.56, 36.19, 36.80, 36.93, 38.17, 41.25, 41.31, 41.38, 44.15, 49.88, 57.45, 58.24, 68.66, 72.39, 181.71.

*3α,6α-Bis(tetrahydropyranyloxy)-24-nor-5β-cholan-23-ol* (**16**). To a solution of **13** (3.0 g, 5.35 mmol) in dry THF (150 mL) LiAlH_4_ (0.99 g, 26.7 mmol) was added portionwise at room temperature. The resulting suspension was stirred at room temperature for 3 h. The reaction was quenched by adding Na_2_SO_4_ 10·H_2_O (15 g) and stirred at room temperature for 1 h. The solid was removed by filtration and the desired alcohol **16** (2.84 g, 5.35 mmol) was obtained from evaporation of the solvent in quantitative yield. ^1^H-NMR (CDCl_3_, 400 MHz) δ: 0.60 (3H, s, 18-*CH_3_*), 0.86–0.87 (3H, m, 19-*CH_3_*), 0.89 (3H, d, *J =* 6.5 Hz, 21-*CH_3_*), 3.33–3.45 (2H, m, C*H_2_*-THP), 3.58 (2H, t, *J =* 7.0 Hz, 23-*CH_2_*), 3.63–3.69 (1H, m, 3-C*H*), 3.85–3.90 (2H, m, C*H_2_*-THP), 3.94–3.99 (1H, m, 6-C*H*), 4.53–4.64 (1H, m, OC*H*-THP), 4.68–4.71 (1H, m, OC*H*-THP).

*3α,6α-Bis(tetrahydropyranyloxy)-24-nor-5β-cholan-23-al* (**17**). To a solution of alcohol **16** (3.5 g, 6.57 mmol) in dry CH_2_Cl_2_ (180 mL) Dess Martin periodinane (4.2 g, 9.9 mmol) was added and the mixture was stirred at room temperature over night. The reaction was quenched by adding NaHCO_3_ss (200 mL) containing five equivalents of Na_2_S_2_O_3_. The biphasic mixture thus obtained was stirred for 30 min, then the phases were separated. The water phase was extracted with CH_2_Cl_2_ (3 × 80 mL) then the combined organic layers were washed with H_2_O (200 mL), brine (200 mL), dried over Na_2_SO_4_ and evaporated under reduced pressure, obtaining the desired aldehyde **17** in quantitative yield (3.5 g, 6.57 mmol). ^1^H-NMR (CDCl_3_, 400 MHz) δ: 0.65 (3H, s, 18-*CH_3_*), 0.87–0.89 (3H, m, 19-*CH_3_*), 0.97 (3H, d, *J =* 6.2 Hz, 21-*CH_3_*), 3.38–3.47 (2H, m, C*H_2_*-THP), 3.55–3.63 (1H, m, 3-C*H*), 3.82–3.92 (2H, m, C*H_2_*-THP), 3.93–4.00 (1H, m, 6-C*H*), 4.53–4.65 (1H, m, OC*H*-THP), 4.69–4.72 (1H, m, OC*H*-THP), 9.72 (1H, s, CO*H*).

*3α,6α-Dihydroxy-24-methyl-5β-homochol-23-en-25-oic acid* (**18**). To a suspension of (1-(ethoxycarbonyl) ethyl)triphenylphosphonium bromide (9.65 gg, 21.8 mmol) (prepared by refluxing ethyl 2-bromo propionate and PPh_3_ in benzene [43]) in dry THF (50 mL) potassium *t-*butoxide (19.8 mL, 1 M in THF) was added and the resulting mixture was stirred at room temperature over night. A solution of the aldehyde **17** (3.5 g, 6.6 mmol) in dry THF (40 mL) was then added and the resulting mixture was refluxed for 1 h. The reaction mixture was allowed to cool at room temperature, poured into H_2_O (100 mL) and extracted with EtOAc (2 × 70mL). The combined organic layers were washed with brine (100 mL), dried over Na_2_SO_4_ and evaporated under reduced pressure. The resulting brown oil was dissolved in 5% HCl in MeOH (100 mL) and stirred at room temperature for 1 h. The solvent was then removed under reduced pressure, the residue was dissolved in H_2_O (120 mL) and extracted with CHCl_3_ (3 × 60 mL). The combined organic layers were washed with H_2_O (60 mL), brine (60 mL), dried over Na_2_SO_4_ and evaporated under reduced pressure. The crude was purified by flash chromatography eluting with petroleum ether/EtOAc from 0 to 20%. The resulting white solid was dissolved in 30 mL of 5% NaOH in MeOH and the resulting mixture was stirred at 60 °C over night. The solvent was evaporated under reduced pressure, the residue was dissolved into H_2_O (100 mL), acidified with 3N HCl and extracted with EtOAc (3 × 60mL). The combined organic layers were washed with H_2_O (100 mL), brine (100 mL), dried over Na_2_SO_4_ and evaporated under reduced pressure to afford the desired acid **18** (1.93 g, 4.62 mmol, 70%) as pure white solid. ^1^H-NMR (MeOD, 400 MHz) δ: 0.73 (3H, s, 18-*CH_3_*), 0.96 (3H, s, 19-C*H_3_*), 0.99 (3H, d, *J =* 6.5 Hz, 21-C*H_3_*), 1.83 (3H, s, 26-C*H_3_*), 3.49–3.57 (1H, m, 3-C*H*), 4.01–4.07 (1H, m, 6-C*H*), 6.85 (1H, t, *J* = 7.0 Hz, 23-C*H*). ^13^C-NMR (MeOD, 100.6 MHz) δ: 12.29, 12.49, 15.24, 19.37, 21.70, 23.89, 25.11, 29.23, 29.79, 30.93, 35.34, 36.00, 36.15, 36.60, 36.73, 37.35, 41.06, 41.09, 43.98, 48.16, 57.36, 57.40, 68.44, 72.18, 129.38, 142.67, 171.40.

*3α,6α-Dihydroxy-24(S)- and -24(R)-methyl-5β-homocholan-25-oic acid* (**6** and **7**). To a degassed solution of the olefin **18** (200 mg, 0.50 mmol) in MeOH (15 mL), PtO_2_ 10% (20 mg) was added and the resulting solution was hydrogenated at 3.5 atm at room temperature for 12 h, to obtain a mixture of C_24_(*S*) and C_24_(*R*) isomer in a 6:4 ratio, as evaluated by HPLC analysis. The mixture was then filtered through Celite, washing the filter with MeOH. The solvent was evaporated under reduced pressure and the crude was purified by medium pressure flash chromatography, affording pure **6** as white solid (90 mg, 0.22 mmol) and an inseparable mixture of **6** and **7** (105 mg, 0.26 mmol). Compound **6**: ^1^H-NMR (MeOD, 400 MHz) δ: 0.72 (3H, s, 18-*CH_3_*), 0.95 (3H, s, 19-C*H_3_*), 0.98 (3H, d, *J =* 6.5 Hz, 21-C*H_3_*), 1.15 (3H, d, *J =* 6.9 Hz, 26-C*H_3_*), 3.51-3.54 (1H, m, 3-C*H*), 4.02-4.05 (1H, m, 6-C*H*). ^13^C-NMR (MeOD, 100.6 MHz) δ: 12.49, 17.41, 19.35, 21.91, 24.10, 25.28, 29.22, 29.98, 31.13, 31.40, 34.45, 35.56, 36.19, 36.80, 36.92 (2×), 37.03, 41.02, 41.31, 41.36, 43.96, 49.85, 57.42, 57.62, 68.65, 72.38, 180.85.

### 3.3. CMC Determination

All the measurements were conducted according to a previously reported procedure [38].

### 3.4. Molecular Modelling

The compound models were built using sketch module of the Maestro 9.3 package and the geometry was optimized adopting the OLPS2005 force field. Each model was submitted to a conformational analysis. The calculation was done with Macromodel 9.9. The conformational search was carried out using the Montecarlo method, and each conformer was minimized using the OPLS2005 force-field. The conformers with an energy difference higher than 5.0 Kcal/mol from the global minimum energy conformation were rejected. Finally, all the redundant conformers showing a root mean square deviation (RMSD) < 0.1 Å (calculated using the heavy atoms) were eliminated. For each compound, the resulting conformers were submitted to a second stage of quantum mechanics geometry optimization using Jaguar 7.9. These calculations were carried out using B3LYP/6-31G** in the solution phase with the PBF solvation model. Only the global minimum conformation for each of the compounds was stored and the relative hydrophilic surfaces were calculated using the Maestro 9.9 package. In particular, hydrophilic surfaces were calculated at 0.8 isovalues to display regions of relatively strong polarity.

## 4. Conclusions

We have reported the synthesis and micellization behaviour of a number of side chain-modified HDCA derivatives. Overall, the collected data depict a clearer scenario on the structure-CMC relationships of BAs unveiling some peculiar properties shared by the molecular shape of BAs. In particular, we have demonstrated the strong effect on the CMC value of the substituent optical configuration at the alpha position to the carboxylic group with the *R*-epimer being more prone to form micelles compared to the corresponding *S* one. As an additional observation, the side chain elongation was found to reduce the different micellization ability between the diverse couple of epimers, as quantitatively confirmed by RMSD analysis. In summary, the results herein presented can be of great utility in combination with model of receptor activity, to guide the development of novel BA-based receptor modulators with improved pharmacokinetic profile and drug-like properties.
